# Individuals with developmental disabilities make their own stylistic contributions to text written with physical facilitation

**DOI:** 10.3389/frcha.2023.1182884

**Published:** 2023-10-31

**Authors:** Giovanni Nicoli, Giulia Pavon, Andy Grayson, Anne Emerson, Michele Cortelazzo, Suvobrata Mitra

**Affiliations:** ^1^ School of Social Sciences, Nottingham Trent University, Nottingham, United Kingdom; ^2^ Faculty of Social Sciences, Nottingham University, Nottingham, United Kingdom; ^3^ Dipartimento di Studi Linguistici e Letterari, Università Degli Studi di Padova, Padua, Italy

**Keywords:** facilitated communication, stylometry, co-authorship, developmental disabilities, authorship attribution

## Abstract

**Introduction:**

For individuals with developmental disabilities (DD) such as autism, Down syndrome, or cerebral palsy, learning to express with language is a two-fold challenge because atypical cognitive capacity is compounded by sensorimotor coordination deficits. One approach to assisting linguistic expression in these individuals is to physically support them, for example, by touching their torso or arm as they type. The neurophysiological mechanism of such motor assistance for linguistic expression is not known, but recently it has been proposed that light touch may reduce the cognitive load associated with the sensorimotor coordination of typing, thereby releasing shared cognitive resources to the task of generating content. Historically, there has been significant controversy over the extent to which the facilitator and not the user authors texts written with touch assistance. User groups and a few researchers have argued that the user can express their thoughts through such techniques, but the prevailing view among researchers is that these texts are entirely the by-products of the facilitators' ideomotor cueing of users' movements. If the user is not a source of the produced text, the only linguistic style detectable in the text should be the facilitator's.

**Methods:**

Here, we use quantitative linguistic analysis to investigate whether DD users typing text with touch assistance exhibit their own stylistic signatures alongside those of their facilitators. In Study 1, we investigate whether the stylometric fingerprints of a set of users are detectable when they are all assisted by the same facilitator. In Study 2, we examine whether the users' stylometric characteristics are retained even when they are assisted by multiple facilitators.

**Results:**

Across both studies, the results show that the users' stylistic signature is detectable alongside that of facilitators. This suggests that the texts generated by DD users withphysical assistance should be viewed as coauthored rather than wholly authored by facilitators via ideomotor processes.

**Discussion:**

The users' stylometric presence in these texts suggests that touch-assistance may serve as a developmental scaffold and should be re-appraised as a teaching aid even where unassisted linguistic expression is an unlikely end goal.

## 1. Introduction

Individuals growing up with a range of developmental disorders face sensorimotor and cognitive deficits that make it difficult to learn a system of communication (1, 2). Augmentative and alternative communication (AAC) systems are designed to build upon existing skills and competencies and ease the learning workload by providing different means or codes through which communicative intentions can be channelled (1, 3). Aided AAC methods employ technological extensions (for example, eye-trackers, motion-sensors, input devices, etran table, and so on) whereas unaided ones (for example, sign language and coded behaviors) do not (3). AAC methods also differ in the communicative code they adopt, which can be orthographical (phonemes associated with orthography), haptic (braille), gestural (sign language) or iconographic/ideographic (based on immediate or indexical similarity, or arbitrary convention) (3, 4). AAC systems may be used to overcome sensory impairment (braille or sign language), fine and gross motor impairment (eye-tracking, etran table), cognitive/linguistic deficits (picture-based systems), or both motor and cognitive challenges (picture-based communication software, rapid prompting method, facilitated communication) (3, 5, 6).

Motor-facilitating AAC devices seek to counter users' physical impairment by exploiting residual motor competences, such as eye-fixations or residual goal-directed index movements, to create text-based communication (3). Cognitive and linguistic facilitation enables users to reduce the workload of phonological word-segmentation (required by a text/keyboard-based communication) and the workload of word reading, lexical access and, in some cases, syntactical and morphological planning, by providing picture-based lexical referents from a closed set of options (3, 5, 6). Combined motor-cognitive facilitation provides a simplification of motor access using technology (for example, eye-tracking), or through the physical support of a facilitator, such as in the rapid prompting method (RPM) or facilitated communication (FC). These methods provide cognitive simplification by reducing the cognitive demands of formulating a full-language expression (e.g., picture-based exchange systems) or by reducing the overall cognitive demands of the task by supporting attention, providing emotional reassurance and reducing the cognitive demands of the co-occurrent motor task while maintaining -text-based communication, as has been suggested for RPM (7, 8) and FC (9).

The development of human-assisted motor-cognitive facilitation systems such as RPM and FC have controversial histories due to concerns over the extent to which the facilitator contributes to the user's communication (10). In the case of FC, for example, where the facilitator assists the user's postural and motor stability by touching their shoulder or holding their arm, it has been suggested that the texts produced are simply by-products of the facilitators' unconscious and ideomotor cueing of the users' movements towards the keyboard (11–15). In this context, the question of authorship has been studied mostly using the message-passing task, in which the user is required to type information about which the facilitator is either unaware or misinformed. As the proportion of messages passed correctly is meagre—(13, 16, 17), except for some partial exceptions (18), the prevailing view is that authorship should not be attributed to FC users (19, 20), and that FC should not be used in formal education (21).

Alternative methods of studying the FC process have suggested a more complex picture of FC users' involvement, such as (22) demonstration of pre-emptive eye-fixations on to-be-typed keys before the typing movements start, or Faure et al's (23) accelerometry study showing that the user's movement towards the keyboard starts before the facilitator's assistive movement. Linguistic analysis of FC users' text have found unexpected or unusual lexical choices (24, 25), linguistic idiosyncrasies (24, 26, 27), spelling errors (26, 28), unusual syntax (25, 26) and differences in terms of MLU (medium length utterance) when compared with texts written by facilitators (27). These results appear to suggest some active involvement of the user in the text production process.

In terms of the properties of the generated text, studies conducted within the EASIEST project (29–35) used hierarchical clustering methods to show that FC users’ texts were stylistically different from those of their facilitator, as indicated by a different ratio in the use of adjective and adverbs (32), an increased number of low-frequency word neologisms and adverbs with the Italian /-mente/ suffix (30, 36), and -frequent use of figures of speech such as anastrophe, “tmesi”, metaphors, and word inversions (31). As pointed out by Saloviita (15), differences in style between FC users' and facilitators' texts does not prove that users are the true authors of the texts they produce. It could be argued that text produced by FC users with the facilitator's assistance might be different in style to text produced by FC users without assistance. This scenario is not testable as users adopt FC because they cannot type independently, but it is consistent with Pennebaker's (37) synergy hypothesis defining the stylistic features of texts produced by multiple authors. Each author involved in the co-creative process is expected to lose their own stylistic fingerprint, creating different stylistic features in the co-created text. Besides, facilitators may simply change their own writing style when involved in the FC process as suggested by Saloviita (15) and Eder (38).

Rather than trying to address stylistic differences between FC users and facilitators when they operated individually, Emerson (39) considered whether the signatures of both might be embedded in the text they produce together. This approach revealed occurrences of lexical choices that could be linked to the user (words used only by the same user with different facilitators) alongside those that were linked to the facilitator (words used by different users only when assisted by the same facilitator). Such results suggest that FC texts are co-constructed by the user and facilitator (24, 40). The plausibility of the co-construction hypothesis depends upon indications of co-authorship at the levels of lexical choice, syntactical patterns (use of function words and word sequences), distribution of morphological markers and phonological/graphemic patterns (through the analysis of short sequence of characters). The potential value of such co-authored text for the communicative, educational, or cognitive and emotional growth of individuals facing the multi-faceted challenges of developmental disabilities (DD) is a separate issue, to which we return in the discussion.

Quantitative methods of linguistic analysis offer several ways to investigate contributions to authorship within corpora of text. Stylometry measures concrete, discrete, non-linear and even non-linguistic (41) textual features to identify authors' “fingerprints” (42). The linguistic fingerprints can then be compared to address authorship attribution issues. Stylometry allows a “distant reading” (43) of texts, enabling the quantification and comparison of broad textual patterns that are unlikely to be consciously manipulable. One approach is to create a simplified model, such as the bag-of-words, whereby texts are considered as lists of words or character n-grams (sequences of characters of different size), sorted by frequency. Each word appearing within the text is a dimension and its frequency the value along that dimension. Casting pieces of text as vectors occupying -locations in a space enables a range of statistical analyses on the stylistic distance between the vectors.

It is very important to be careful with the term “authorship” in this context. The attribution of authorship using stylometric analysis concerns patterns of language use. Stylometry cannot be used to query whether the thoughts expressed in the text are the author's own. This, however, is the sense of the term “authorship” at the core of controversies about touch-assisted typing by individuals with DD. Stylometry applied to text written using FC cannot address whether the thoughts expressed in the text are the user's or the facilitator's. It can only detect the presence of stylistic patterns attributable to each. As such, “authorship” and “co-authorship” are used in this article in the stylometric sense, encompassing the syntactic, lexical, morphological, and phonological patterning of text.

One analysis approach uses unsupervised learning algorithms to calculate the distance between vectors. The shorter the distance, the closer the texts in stylistic features (in terms of lexical, morphological, or syntactic choices). Several algorithms have been proposed for calculating the distance between texts, starting with Burrows' delta or “classical delta” (44, 45) to “cosine delta distance” (46, 47), which computes a cosine similarity between a matrix of values normalized (z-scored) to minimize matrix size. Once the texts are organized in a distance table (with respect to their distances to other texts), their similarities can be expressed in multiple ways. Hierarchical clustering analysis, for example, displays texts in a dendrogram that progressively pairs them based on similarities. Thus, texts occupying the same leaf are highly similar. Another method of expressing stylistic relationships between texts is the bootstrap consensus network (48), which graphically displays stylistic similarities through linkages of varying thickness.

These stylometry methods can be valuable in investigating the issue of authorship of texts generated through AAC techniques such as FC as they provide a time-extended perspective on the text construction process as it operates naturally, without the insertion of experimental artifacts. The analysis of text generated over long periods of time, while the user and their partnership with facilitators evolve, provide a stronger test of individual influences than snapshot methods using arbitrary tasks in which the user possibly is not motivationally invested. Linguistic analysis may also address authorship questions at multiple levels: lexical, by investigating lexical choices, syntactical, by focusing on the use of function words and word sequences, morphological, by observing the distribution of morphological markers, and phonological/graphemic, by considering character n-gram patterns.

The focus of the present paper is to conduct multi-level quantitative linguistic analyses of corpora of text generated by multiple FC users with the same facilitator (Study 1), and text produced by multiple users, each with multiple facilitators (Study 2), to determine whether, or to what extent, the FC users' stylistic signature can be detected alongside that of their facilitators. In both studies, we start from the maximally sceptical position that the facilitator is the sole ordering agent, and that text produced using FC is not objectively attributable to any source other than the facilitator. It should not be possible, then, to find unique stylistic fingerprints associated with specific FC users. We use unsupervised machine-learning methods, particularly cluster analysis, to test whether the stylistic distance between texts (based on intrinsic stylistic features) is governed solely by the characteristics of the facilitator. We first describe the characteristics of the texts that we analysed, and then present the two studies.

## 2. The corpora

The texts analyzed in the present studies were collected from two FC centers in Italy. The texts are therefore in Italian and have not been used previously in research. To accumulate a sufficient amount of data for each FC user, we collected pre-existing texts written over the past two decades in the course of each center's usual practice. For the purpose of this project, we asked each center to forward all the pre-existing texts produced by their clients that they had stored in their databases. The centers gave written consent for us to use their data in our analyses under the condition that our reports would be fully anonymized at the levels of the users, facilitators, and the centers. As the present work only reports fully anonymized analysis of pre-existing data, and we obtained consent from the holders of the data, we did not require ethical approval for these analyses.

In Center 1 and Center 2, the texts were stored in a specific folder named after each participant, each.docx file within each folder representing the text output of one FC session, held on a weekly basis. Most of the texts were in a dialogue form, therefore they contained lines from the user and lines from the facilitator (highlighted in caps lock). Each document identified the facilitator who assisted the FC session reported in it.

The texts were then pre-processed in the following steps.

**Figure 1:**
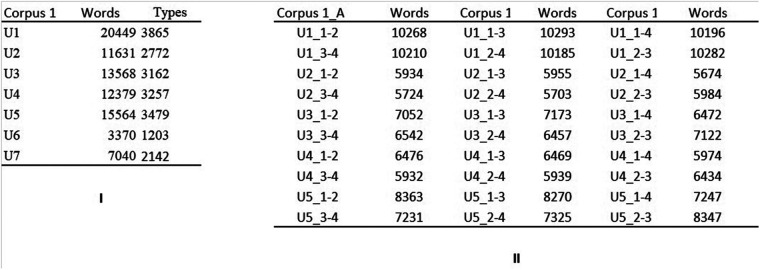
Corpora from center 1. (**I**) Corpus_1. Texts collected from center 1. All texts were written with a single facilitator. (**II**) Corpus 1_A, corpus 1_B, and corpus 1_C. The text production of each user, after being divided in four homogeneous chunks, is merged in three different ways. In center1_A, the first chunk is merged with the second chunk, and the third and the fourth chunks are merged together. In center1_B, the first chunk is merged with the third, and the second and the fourth chunks are merged together. In center1_C the first chunk is merged with the fourth, and the second and the third chunks are merged together.

**Figure 2:**
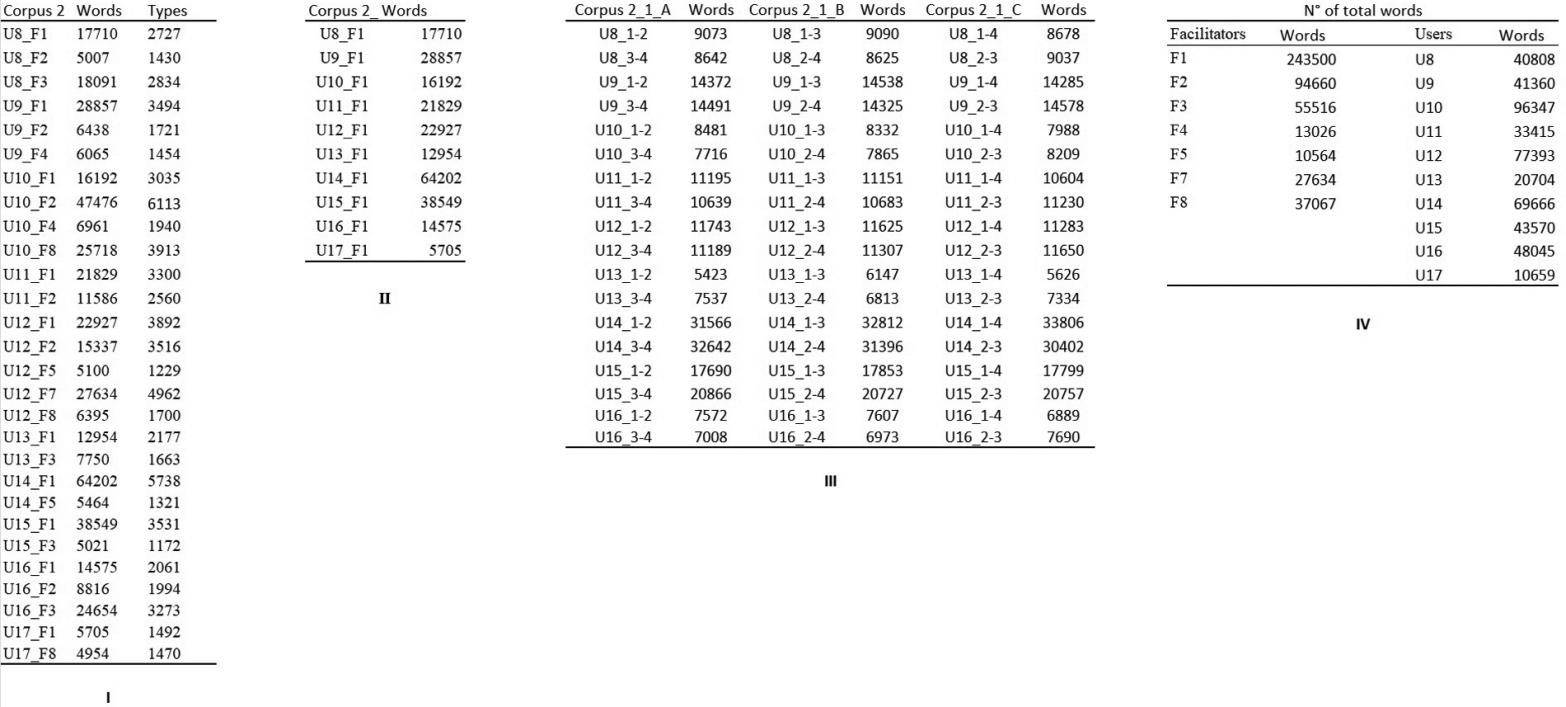
Corpora from center 2. (**I**) Corpus_2. Texts collected from center 2. Users were assisted by multiple facilitators. (**II**) Corpus 2_1. Texts from corpus 2 written solely with F1. (**III**) Corpus 2_1_A, corpus 2_1_B, and corpus 2_1_C The text production of each user, after being divided into four homogeneous chunks, is merged in three different ways. In corpus 2_1_A, the first chunk is merged with the second chunk, and the third and the fourth are merged. In corpus 2_1_B, the first chunk is merged with the third, and the second and the fourth chunks are merged together. In corpus 2_1_C, the first chunk is merged with the fourth, and the second and the third are merged together. (**IV**) A summary of the total words available for each user (independently of the facilitator) and each facilitator (independently of the user). Note that nearly half of the words collected in center 2 were typed with the assistance of facilitator 1 (F1).

**Figure 3:**
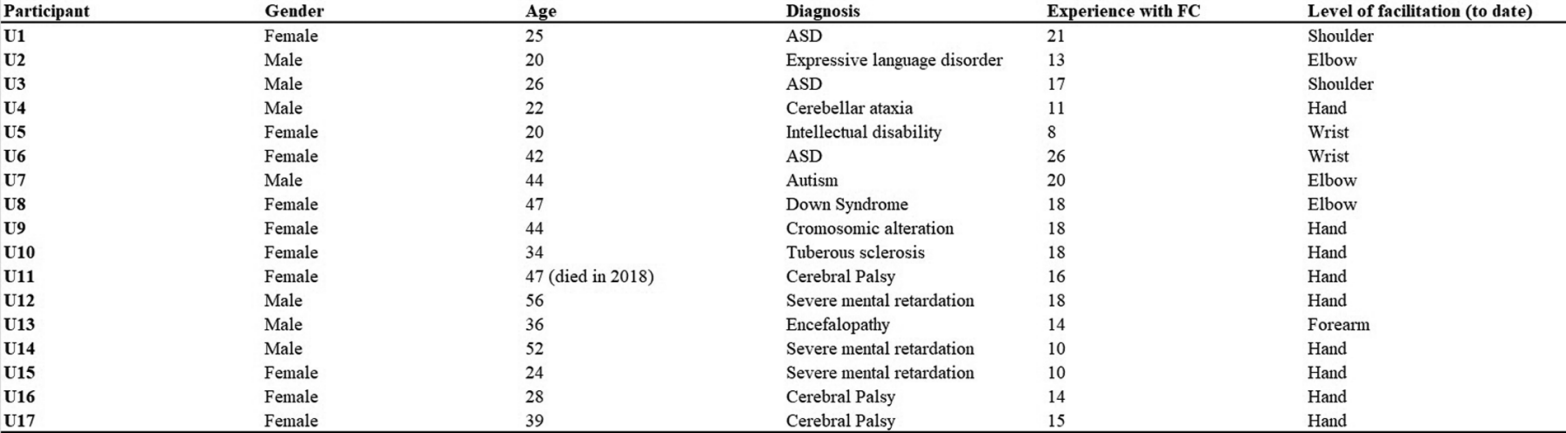
Description of FC users. For each FC user, gender, age, diagnosis, years of FC use and the actual level of facilitation are shown. Users U1–U7 came from center 1, and users U8–U17 from center 2. All the users reported in the table could not communicate independently through writing. Note that the terminology that was used in the original diagnoses is retained in this figure.

First, the texts were divided by user-facilitator pairings. All the users from Center 1 wrote with the same facilitator, but the users from Center 2 were assisted by multiple facilitators. Second, the users' lines were automatically separated from facilitators' lines using a python script. Any references to the users' or facilitators' names were then removed from each file. Finally, all the files for each user-facilitator pairing were grouped into a unique.txt file and coded in UTF-8 to allow R software to run the analyses on the files. These operations created Corpus 1 for Center 1 and Corpus 2 for Center 2 (see Figures 1I, 2I). Corpus 1 had 7 participants (3 Female, 4 Male; Age: 19–44 yrs, mean = 25.8; >10 yrs since FC adoption), and Corpus 2 had 10 participants (7 Female, 3 Male; Age: 21–54 yrs, mean = 38.9; > 10 yrs since FC adoption). All the users involved in this study could not communicate independently through writing and, in some cases, they were diagnosed with mild to severe intellectual disability. The users' information is shown in Figure 3. The users' names were coded by assigning to each user a number (U1, U2, and so on). For Corpus 2, the facilitators' names were coded with the letter “F” followed by a progressive number (F1, F2 and so on). The users' and facilitators' names were separated by an underscore (“_”). According to the needs of the studies, these files were then assembled into different corpora created specifically to address the research questions (see the Materials section of each study).

Before addressing these texts quantitatively in Study 1 and Study 2, we consider both corpora in their entirety, focusing on the global characteristics of the writings of FC users. Corpus 1 is composed of 84,001 occurrences (tokens, the number of total words used in the corpus) resulting from the use of 10,552 words (types, number of different forms that appears in the corpus). Corpus 2 consists of 481,228 occurrences (tokens) resulting from the use of 20,021 words (types).

According to Bernardi (29), the linguistic production resulting from a process of facilitated communication presents some peculiarities that separate it from neurotypical communication. Some characteristics appear to have nothing idiosyncratic or individual (31) about them, and appear instead to be a trait common to the population who write with FC. The peculiarities of the (Italian) language of facilitated communication, explored in Cortelazzo (30), Benelli and Cemin (36) and Di Benedetto (31) can be summarized as follows: (1) the presence of a varied and non-repeating lexicon, with the presence of numerous Hapax legomena (words that appear- just once in the corpus), (2) the occurrence of uncommon words of the Italian language, (3) the use of common words isolated from their context of natural occurrence, (4) the presence of words that do not exist in the Italian language but are possible, (5) frequent use of forms with the prefix / in /, (6) the intensified use of adverbs in / -mente /, (7) the presence of marked syntactic structures alongside unmarked syntactic structures, and (8) strong incidence of left-side dislocations (focalization of a word by putting it first in syntactical construction).

The texts of Corpus 1 and 2 have many features in common with the texts of the EASIEST project (2008). Corpus 1 and 2 exhibit a non-repetitive and rich lexicon, and also behave in a similar way to the texts used in Bernardi (29) with regard to uncommonly used word forms. The analysis with respect to the frequency of the different words, conducted with the CoLFIS (49) software, shows that there are numerous uncommon forms within the corpus. Thus, among the words of uncommon use, as in those of common use, the presence of numerous forms introduced by the in- negative prefix is confirmed. As described in Bernardi (29), it is also possible to identify some words within Corpus 1 and 2 that are not part of the Italian language lexicon but which are possible as they are constructed according to the rules of word formation. Among these, it is possible to notice that the adjective formation from a nominal base through the suffix /-oso/ is very frequent and used correctly in most cases to indicate the presence and abundance of the quality expressed by the name from which it derives. Similarly, we noted the tendency to form denominal verbs (for example “vacanzare”) or de-aggettival (for example “tristeggiare”). Finally, the production of new possible words is productively originated through the creation of adverbs in /-mente/: in the corpus we can in fact observe forms such as “narcisamente” (narcissistically), not present in the Italian vocabulary.

From a syntactical standpoint, we noticed that in Corpus 1 and 2 there are sentences with a marked syntactic structure, although less frequent compared to the texts described in Bernardi (29). It is possible to observe constructions that are freed from the SVO structure through the anticipation of the second argument or the postposition of the predicate (see examples 1–4 below), by reversing the noun adjective order (examples 5, 6), or by avoiding articles and other grammatical elements (examples 7,8,9). However, the incidence of these marked structures is lower than that described in the EASIEST research. These could be the sign of a higher editorial intervention from the facilitators or the result of a process of style development or teaching within each Center. Examples:

1. “suoni buoni faccio” (U15_F1) [I make good sounds]
2. “forte molto grande mi sembra” (U14_F5) [It seems very big and strong]
3. “bravi e testardi operatori ho trovato” (U17_F8) [I found good and stubborn operators]
4. “io donna silenziosa sono” (U9_F2) [I am a silent woman]
5. “sono come vulcanosi monti ormai svuotati che hanno bruciato le loro emozioni” (U9_F4) [I am like vulcanous but emptied mountains that have burned their emotions]
6. “forte senso di piacere nei rigidi muscoli” (U11_F2) [strong sense of pleasure in rigid muscles]
7. “io dico che importante problema si verifica” (U12_F5) [I say that it is verifying an important problem]
8. “rabbioso momento interno mi ha colpito” (U13_F3) [ a rabid internal moment hit me]
9. “gambe non aiutano ma mani si” (U16_F2) [legs do not help, but hands do]

## 3. Study 1

In this study, we investigate the stylistic characteristics of text produced over 10 years by sets of FC users each working with the same facilitator. We collect and separately analyse, using identical methods, texts from two independent FC centers in Italy. The users in each center produced their texts with a single facilitator. Our null hypothesis is that texts from each center carry a single stylistic influence—the facilitator. As the FC user is not a significant linguistic agent in this view, texts produced by an individual user are not expected to be more similar to other texts by the same user than to texts by other users. We use unsupervised machine-learning techniques to carry out this similarity analysis. To allow user-to-user comparison, we split each user's text into two chunks of equal length in three different ways. We then measure the stylistic distance between texts and display them on cluster dendrograms. If the facilitator is the sole stylistic agent at each center, the clusters should exhibit no user-related grouping, with texts by the same user unlikely to be paired at the leaf level of the dendrogram.

### 3.1. Materials

As previously introduced, Corpus 1 consisted of seven users' texts written with a single facilitator. From Corpus 2, texts that were written with Facilitator 1 (who was the facilitator most represented in the corpus) were selected to create Corpus 2_1 (Figure 2II), which consisted of 10 users' texts. Thus, the included users from both Corpus 1 and 2 had written with a single facilitator at their Center, and each user's text was more than 5,000 words in length (50).

For cluster analysis, each text was split into 4 equal-length fragments that were subsequently assembled in three different ways to create two equal-length chunks for each text. The chunking was done using different combinations of text fragments, and the analyses repeated for each method of chunking, so that the results could not be dependent upon the accident of a specific chunking approach. First, the texts were merged chronologically (thus, fragment 1 and fragment 2 created chunk 1–2, and fragment 3 and fragment 4 created chunk 3–4). This constituted Corpus 1_A and Corpus 2_1_A (recall that Corpus 2_1 contained texts from Center 2 that were written with facilitator 1). Second, chunks were created by joining fragments 1 and 3 (chunk 1–3) and fragments 2 and 4 (chunk 2–4). This created Corpus 1_B and Corpus 2_1_B. Finally, fragments 1 and 4 were merged to give chunk 1–4 and fragments 2 and 3 gave chunk 2–3. The results of these operations were Corpus1_C and Corpus 2_1_C. As seen in Figures 1I, 2I, the division of U7 and U17's texts would result in chunks of less than 5,000 words. These users were therefore not included for cluster analysis. The texts were named after the user's code and a numerical code referring to the merged chunks (for example, U1_1–2 signified chunks 1 and 2 of U1 merged). The corpora are displayed in Figure 1II for Center 1 and Figure 2III for Center 2.

### 3.2. Methods

Corpus1_* and Corpus 2_1_* were analyzed separately using the clustering method implemented within Stylo for R (50). The analysis was performed based on the first 1,000 most frequent words and on the first 1,000 most frequent character-trigrams. Textual distance was calculated using cosine delta distance (46).

### 3.3. Results

**Figure 4:**
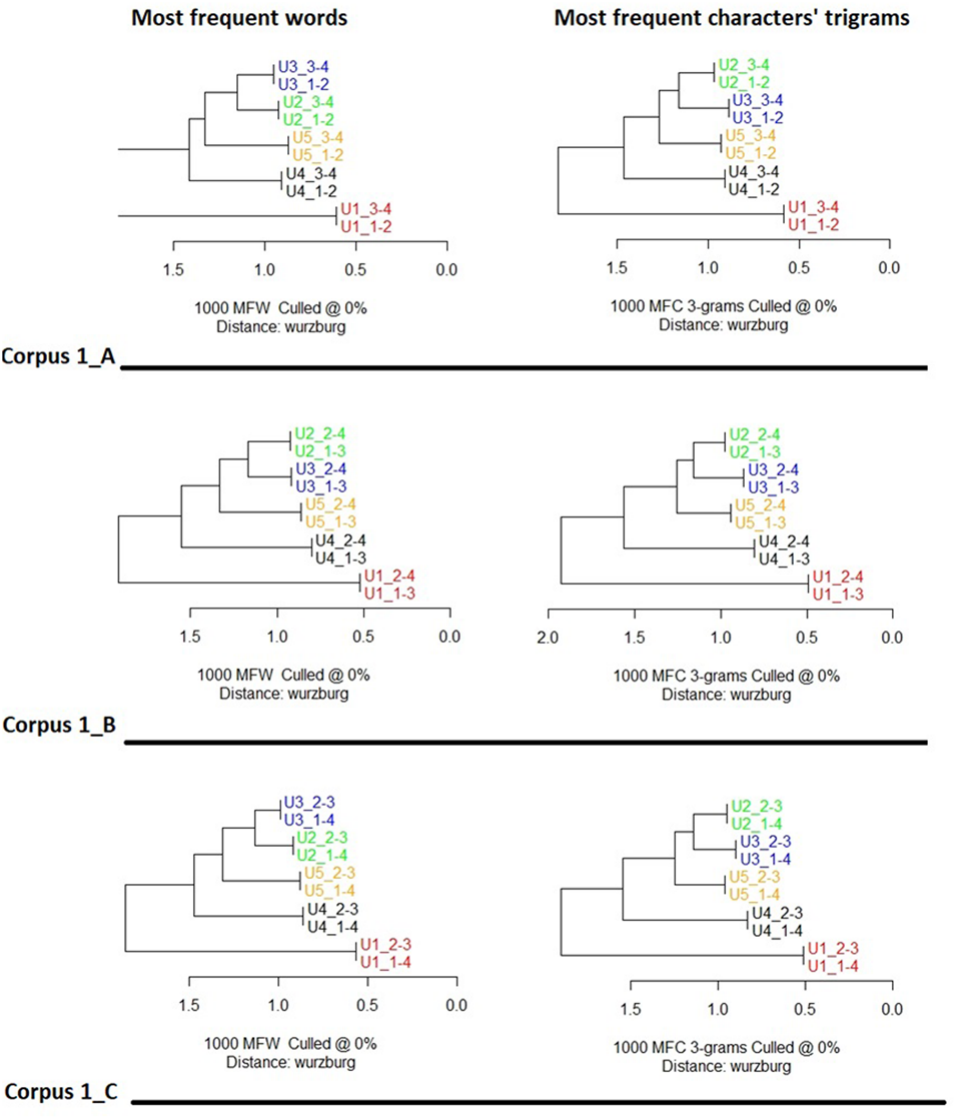
Cluster analysis of texts from corpus 1_A, corpus 1_B and corpus 1_C. Features’ selection 1,000 most frequent words (left column) and 1,000 most frequent characters’ trigrams (right column). Distance: cosine delta. Texts were firstly divided into 4 quarters, then reassembled in three different ways. The code following the underscore indicates the merged quarter. Thus U3_1–2 indicates the first and second texts’ quarters of participant 3 merged together. In all conditions and features’ selection texts of the same user are systematically, paired together at dendrograms’ leaves level. U1 occupy singularly one of the two major branches, opposed to all the other users.

**Figure 5:**
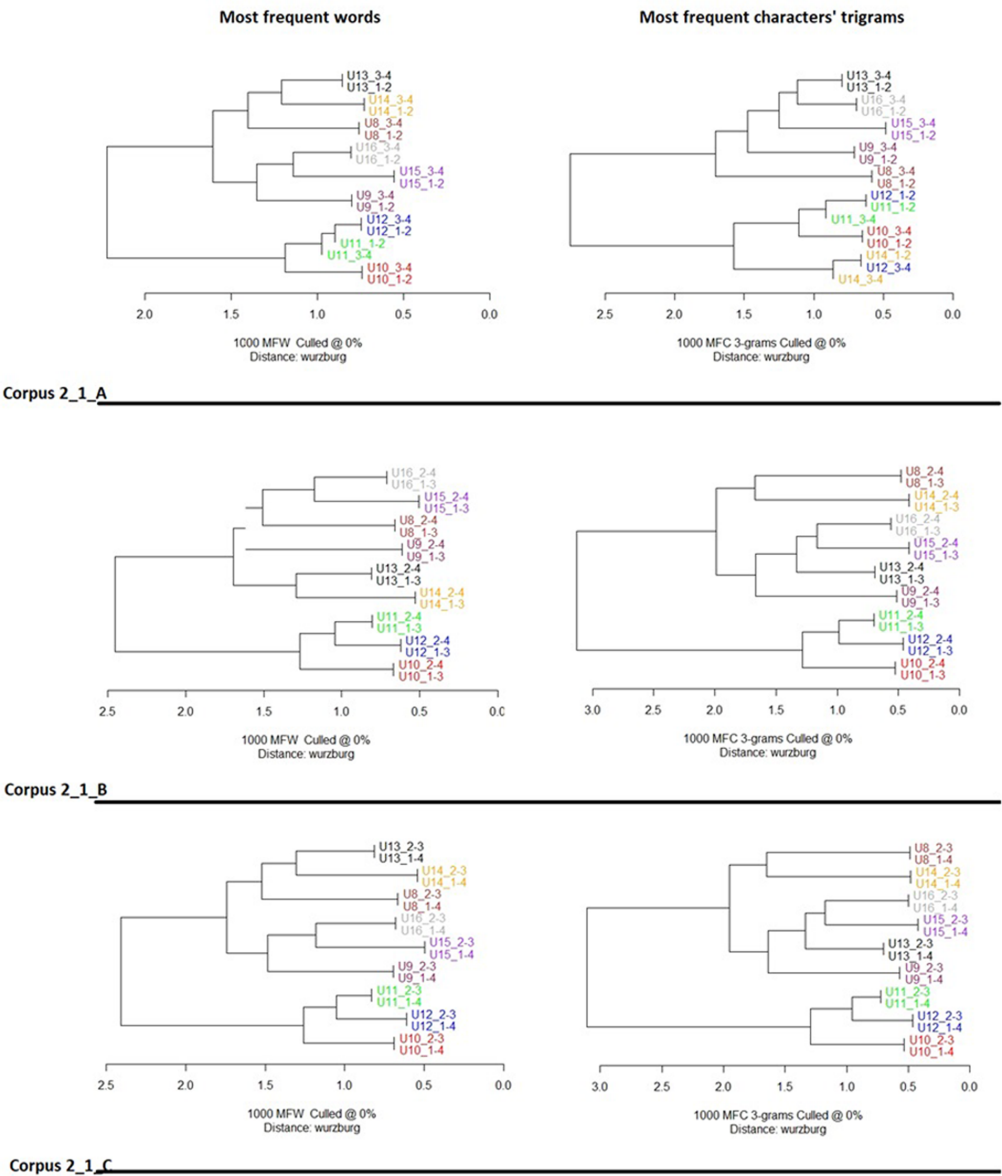
Cluster analysis of texts from corpora corpus 2_1_A, corpus 2_1_B and corpus 2_1_C. Features’ selection 1,000 most frequent words (left column) and 1,000 most frequent characters’ trigrams (right column). Distance: cosine delta. Texts were firstly divided into 4 quarters, then reassembled in three different ways. The code following the underscore indicates the merged quarter. Thus U13_2–3 indicates the second and the third texts’ quarters of U13 merged together. In all conditions and features’ selection texts of the same user are systematically paired together at dendrograms’ leaves level, with the sole exception of corpus 2_1_A characters’ trigrams where participant U12 is paired once with U11, once with U14. Two major users’ groups are displayed. U13, U14, U15, U16, and U8 on the one side, U9, U11, U12, and U10 on the other.

The cluster analysis (Figures 4, 5) showed that texts by the same user consistently produced the closest pairing. In the case of Corpus 1_* (Figure 4), whether using the 1,000 most frequent words or character-trigrams, the closest pairings were always of texts by the same user. This was also the case for Corpus 2_1_* (Figure 5) using words, but there was one notable exception in the analysis by character-trigrams. In one of the three ways of splitting users' text (Corpus2_1_A, where the first two quarters of each user's text were separated from the last two quarters), one of U12's chunks (U12_1–2) was grouped with U11's texts, and the other (U12_3–4) was grouped with U14's texts. This suggests the possibility that U12's style is similar to U11 and U14.

It is also interesting to note the pair relationships across the dendrograms. In the case of Corpus 1_* (Figure 4), U1 is consistently on the lowest, most distant branch. In the upper branch, U2 and U3 are grouped the closest, followed by U5 and U4. This pattern of similarity between users is stable across all three ways of separating their texts and analysis by the most used words or character-trigrams. In the case of Corpus 2_1_* (Figure 5), U10, U11 and U12 always occupy the inferior major branch, separated from all the other users. U15 and U16 are often paired together, as are U13 and U14.

### 3.4. Summary

The study set out with the assumption that the facilitator is the sole stylistic influence on each of the two corpora. If this was the case, the cluster analysis results would not be expected to show any pattern of stylistic similarities between texts. This would apply both to the similarity between chunks of text by the same user and to the stylistic proximity of texts across users. The cluster analysis results were not consistent with the starting assumption. As is clear from Figures 4, 5, chunks of text by the same user were almost perfectly grouped together, suggesting that the unsupervised machine-learning algorithm could reliably detect the unique stylistic signature of most of the users. There were also clear and stable patterns of differential similarity between users, which is also not consistent with the starting hypothesis.

## 4. Study 2

In Study 1, we considered texts from multiple users who worked with the same facilitator. For both the FC centers from which we collected texts, we tested and rejected the hypothesis that the facilitator was the sole detectable stylistic influence on the texts produced by users at each center. In this study, we analyze texts produced over a period of 10 years by FC users who were assisted by a pool of facilitators, each user working with multiple facilitators. From Center 2 (introduced in Study 1), we included texts of users who wrote consistently with at least two facilitators. As in Study 1, our starting hypothesis was that only the facilitators' stylistic characteristics should be distinguishable in the corpus. As, in this view, the users have no stylistic signatures of their own, texts by different users should group only according to the facilitators who assisted their production. Equally, texts by the same user with different facilitators should also group only with the facilitators. As in Study 1, we use unsupervised machine-learning to organize the texts by stylistic similarity. We consider all the texts that were produced by each user-facilitator pairing and investigate the extent to which texts group according to the facilitator. If the facilitators are the only contributors to the stylistic characteristics of these texts, then we should not see any indications of similarity between texts by the same user.

### 4.1. Materials

For this study, we used texts from Corpus 2 (Figure 2I) as this corpus satisfied the conditions we wanted to test. As many FC users at Center 2 have typed texts with the assistance of multiple facilitators, it was possible to consider texts of more than 5,000 words written by FC users who had written consistently (>5,000 words) with at least two different facilitators. Corpus 2 contains 28 pieces written by 10 FC users with 7 different facilitators. As is clear from Figure 2I, texts for each user-facilitator pairing were longer than 5,000 words, but the sizes were not balanced. The distribution of facilitators and the number of words typed with their assistance was uneven as well. F1 assisted all ten users (243,500 words), F2 six users (94,660 words), F3 four users (55,516 words), and so on.

### 4.2. Methods

The key question in this study is: do texts written by users with the assistance of multiple facilitators show that the only detectable stylistic signature is that of the facilitator? This question was addressed through two methods, reported in Study 2A and Study 2B. In study 2A, Corpus 2, was analyzed using the hierarchical clustering mechanism implemented in Stylo for R (50). The analysis was conducted on both the 1,000 most frequent words and the 1,000 most frequent character-trigrams. Cosine delta distance was adopted as the measure of distance (46, 47). The cluster analysis returned a distance table where, for each text, its distance from all the other texts involved in the analysis is computed. Since the analyses by words and trigrams produced very similar results, we have reported the analysis by words only.

In study 2B, Corpus 2 was analyzed using the bootstrap consensus network method implemented in Stylo for R (50). The bootstrap consensus network was used to highlight all the relationships existing between texts that could not be visualized in the dendrogram, nor quantified by distance tables. The bootstrap consensus network allows a multiple and progressive evaluation of the corpus using different sets of features (38), in this context vectors of most frequent words ranging from 100 up to 5,000. Again, this analysis conducted with character bigrams and trigrams yielded very similar results, and so are not separately reported. Cosine delta distance was used to compute text similarity, as it is known to provide highly reliable results (46, 47).

### 4.3. Study 2a: cluster analysis

**Figure 6:**
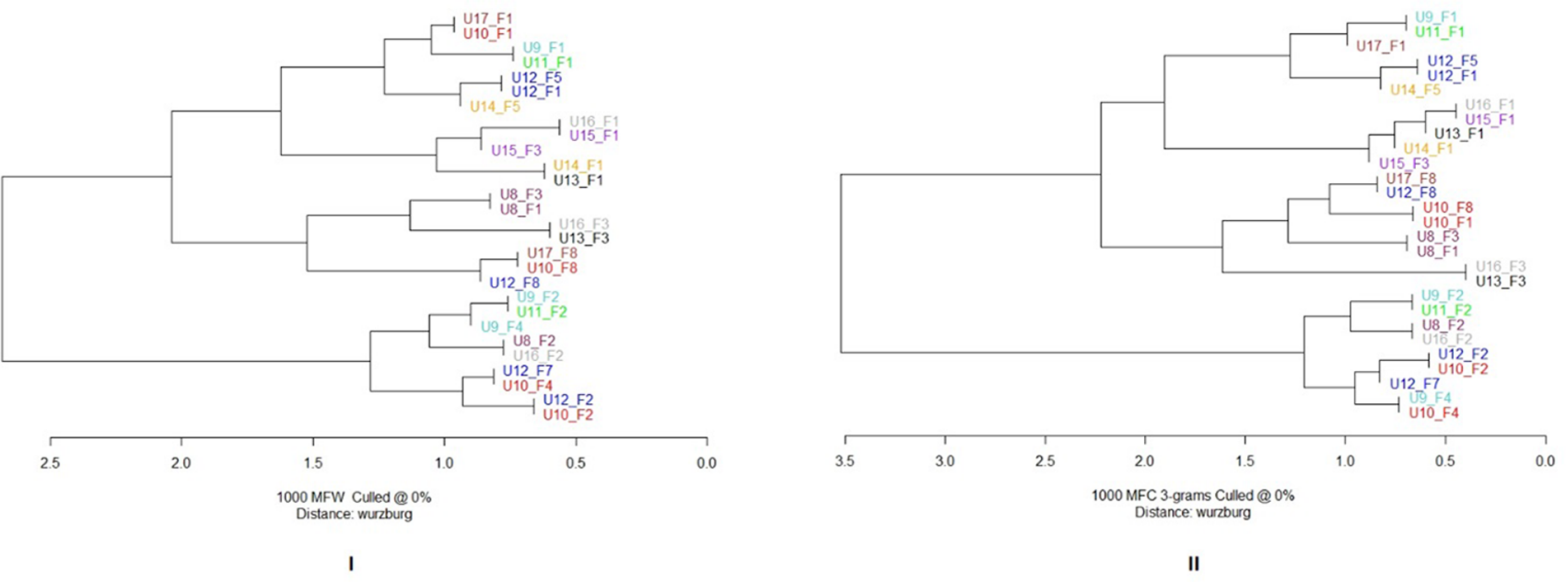
Corpus 2 cluster analysis. Features’ selection: 1,000 most frequent words (**I**) and 1,000 most frequent characters trigrams (**II**) Distance: Cosine Delta. Clustering ratio seems to be facilitator dependent. Texts written with the same facilitator are, in fact, grouped together. For example, texts written with facilitator 1 occupy mainly the upper branch of the dendrogram, while texts written with 2 occupy the lower one.

When texts by each user-facilitator pairing are considered, texts mainly group according to the facilitator rather than the user (Figure 6). However, the distance table associated with this cluster analysis showed patterns that are not seen in the dendrograms. Since words and trigrams distance tables provide similar results, we concentrated just on the words analysis.

**Figure 7:**
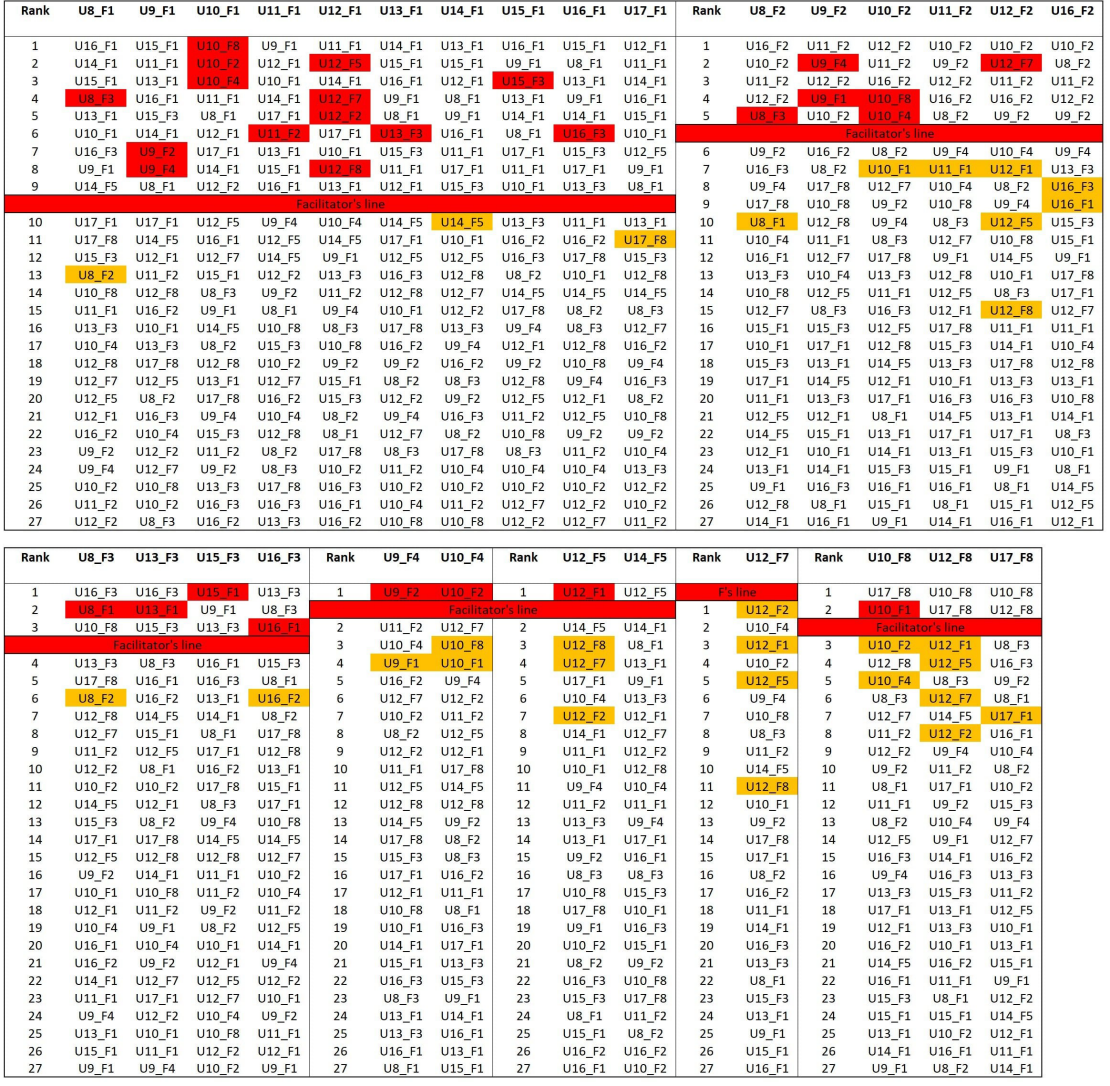
Corpus 2 cluster analysis: distance table. 1,000 most frequent word. Cosine delta distance. Texts are grouped by the facilitator shared, thus the first 10 colums are occupied by those texts written with facilitator 1. Facilitators are cipher coded, users are alphabetically coded. Each row represents a rank position. Higher the rank, stylistically closer the texts. For example, considering U8_F1 in Figure 7 its closest texts are U16_F1, U14_F1, U15_F1 and U8_F3. For U9_F1, U15_F1, U11_F1, U13_F1 and U16_F1 occupy the first four rank position. The thick red line (named Facilitator's line) represent the line above which we should expect to find just those texts that share the same facilitator expressed at the top of the column, according to the hypothesis that facilitators are the sole source of text production. For F1 it is positioned between position 9 and 10, as F1 assisted 10 different users. Texts coded in red refer to texts of the same user (of the one expressed by each column) that rank above the red line. In orange texts that belong to the same user that rank below the red line. 27 (words) and 28 (trigrams) texts are red colored; therefore they have broken the red line. 22 (words) and 23 (trigrams) texts are ranked in the first three rank position, indicating clear user-driven similarity. 8 users out of 10 has at least one text that classify in the top three rank position.

The ranking of each text was analyzed to look for patterns of similarities between texts. If *n* is the number of texts written with the same facilitator, and the facilitator is the only stylistic source in these texts, the distance table should place in the first *n*-1 positions only those texts that shared the same facilitator. In the case of the user-facilitator pair U8_F1, for example, as facilitator 1 assisted in 10 texts, the first nine positions of similarity (i.e., rows in the distance table) should be occupied by the other texts written with facilitator 1 (that is, U9_F1, U10_ F1, U11_ F1, U12_ F1, U13_ F1, U14_ F1, U15_ F1, U16_ F1, and U17_ F1). The *n*-1 value for each facilitator (reflecting the number of users they assisted) is graphically displayed in the Figure 7 by a thick red line (*n *= 10 for F1, *n *= 6 for F2, *n *= 4 for F3, *n *= 2 for F4, and *n *= 3 for F8; F7 worked with only one user).

If the users made no stylistic contribution, their texts should rank randomly below the *n* value for each facilitator. This red line represents the landmark we refer to in our observations. Texts of the same user (expressed by each column) that rank above the red line, are colored red. Similarly, texts that rank below the red line are reported in orange if they belong to the user expressed by the column. The analysis of texts that rank below the red line is particularly interesting for those facilitators that are less represented (as F7). In those cases, since a smaller number of texts could rank above the red line, the ranking of texts that come right below is worth noting.

As we can see from distance tables (Figure 7), 27 texts breach the red line, which means that the users' stylistic contribution in 27 out of 60 cases breach the facilitator-influence barrier, contrary to what is expected from the null hypothesis. Moreover, in 22 cases, the user's texts classify in the first three rank positions, on 32 in the first 5 rank positions, and on 54 occasions out of 60 in the first 10 rank positions. On 11 occasions, texts of different users and facilitators also breach the red line. In most of these cases, the users are the same as the ones that occur in the first rank positions. Consider for example U8_ F1. In the first two rank positions, we find U16_ F1 and U14_ F1. Then at rank 7 and 9 we find respectively U16_ F3 and U14_ F5. Since U8_ F1 is very similar to U16_ F1 and U14_ F1, its similarity with U16_ F3 and U14_ F5 may be due to inter-user stylistic similarity; texts representing the same user but not the same facilitator (i.e., not the one expressed by the column) that rank above the red line might indicate inter-user similarities, as between U8 and U16, that are not determined by the facilitator's influence.

**Figure 8:**
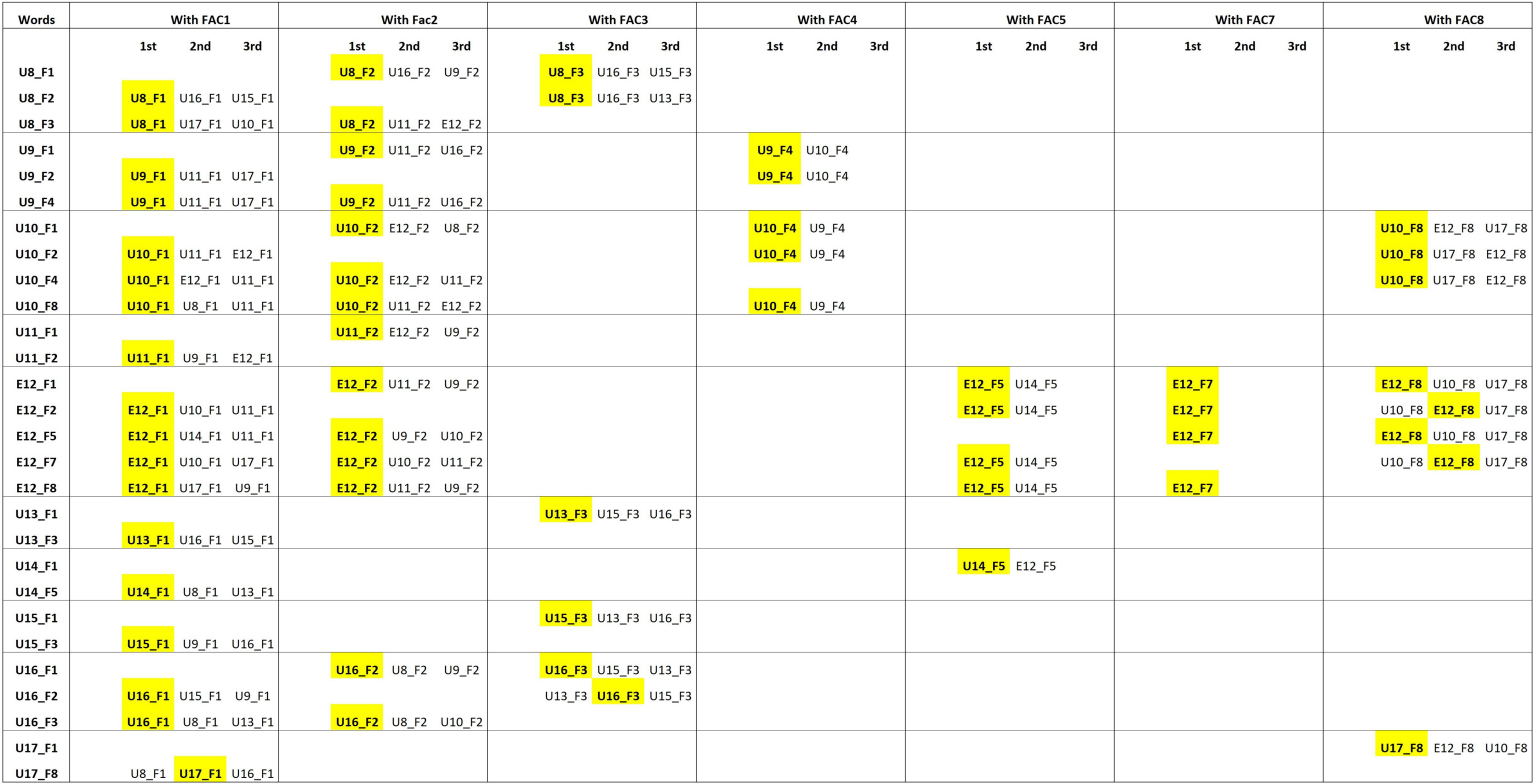
Corpus 2 cluster analysis: texts’ rank positons according to the facilitator they share, in words analysis. For each user-facilitator pair, the first three rank positions of texts grouped by a shared facilitator are displayed. Let US consider U8_1; since U8 wrote with FAC2 and FAC3, the ranking order of texts written with FAC2 and FAC3 relative to U8_1 is reported. If no user contribution is expected, we should not find consistent ranking patterns. Texts coded in yellow refer to texts that belong to the same user. 60 texts out of 60 rank at least in second position. 56/60 texts classify in the first rank position.

Another aspect that is worth noting is that texts of the same user (i.e., the one expressed by the column) rank above all the other texts written with the same facilitator (56/60, see Figure 8). Consider U8_ F1 as an example. U8_ F3 is the first text written with F3 in the ranking; also, U8_ F2 is the first text written with F2 that appears in the distance table relative to U8_ F1. This happens systematically for all texts' relationships and shows that it is the user's contribution, rather than the facilitator's one, that determines the similarity. If no stylistic contribution is made by the user, these consistent and systematic patterns of rankings should not occur; texts by the same user written with different facilitators should rank randomly.

Texts of both different users and facilitators that appear below the red line, do not show a clear and unique pattern of interpretation. The unbalanced nature of the corpus does not allow for direct and exhaustive considerations of the different relationships that are consolidated in the corpus. For that, we would need a 10 × 10 corpus (10 users all assisted by 10 facilitators). However, we can observe some general patterns in terms of distance: inter-facilitator similarities (F1 appears similar to F3 and distant from F2), inter-user similarities (U8 is similar to U16, U9 to U11, U10 to U12, U11 to U9, U10 and U12; U12 to U14 and U10; U13 to U15 and U16; U14 to U12 and U13; U15 to U13, U9 and U16; U16 to U8, U15, and U13), and user to facilitator similarities (U8_F3 and U16_F3 to F8, U9_F4 to F2).

The rank analysis of texts clearly shows how often texts that either share the user or the facilitator occupy higher rank positions. To quantitatively reinforce the rank analysis results, we conducted a set of statistical analyses on the underlying distances between texts resulting from Stylo calculations. Distance values refer to the most frequent words analysis.

In order to statistically address the data, we differentiated five groups of distance values based on the relationships existing between texts. One group (IU) refers to the distance values between texts that share the same user. The second group (FU) refers to the distance values observed between texts that share the same facilitator and users who have stylistic similarities (see inter-user similarities in Study 1 and the cluster analysis in Study 2; inter-user similarities are also summarized in Table 1). The third group (F) refers to distance values found between texts that share the facilitator, but the users do not have stylistic similarities. The fourth group (RU) includes texts that do not share the facilitator but have users with stylistic similarities. Finally, the fifth group (NR) contains texts that do not share facilitator or users with stylistic similarities. No more than two relationships were considered for each user. Table 2 below summarizes the different groups, the number of texts considered within each group and the mean distance value.

**Table 1:** Inter-user stylistic similarities.

User	User with Similar stylistic fingerprint	
U8	U16	U14
U9	U11	
U10	U11	U12
U11	U10	U12
U12	U10	U11
U13	U14	U8
U14	U13	U8
U15	U16	
U16	U8	U14
U17	U8	U16

These relationships were extracted from the cluster analyses described in study 1 and study 2. Similarities with up to two users were assigned. As a result, some of the relationships are not mutual.

**Table 2:** One sample *t*-test.

	One sample *T*-test							
	Nr of distance values	Mean (SD)	*μ*1	Test result	*p*-value	*μ*2	Test result	*p-*value
FU	33	0.79 (0.09)	1	1	0,00000	1.04	1	0.00000
F	109	0.89 (0.12)	1	1	0,00000	1.04	1	0.00000
IU	60	0.89 (0.10)	1	1	0,00000	1.04	1	0.00000
RU	117	1.04 (0.07)	1	1	0,00000	1.04	0	0.37095
NR	437	1.10 (0.08)	1	1	0,00000	1.04	1	0.00000
	*μ*1 = neutral distance value							
	*μ*2 = average distance value							

The *t*-test was run twice, with two different values for μ. The value of *μ* = 1 was chosen as a neutral distance value, since cosine delta distance can assume values between 0 and 2, with 0 referring to the highest level of similarity and 2 to highest level of distance. The value of *μ* = 1.04 was chosen in order to detect differences from the average of the observed distance values. Texts that share either the same user or the same facilitator have distance values significantly lower than both *μ*-values. FU refers to texts that share the same facilitator and users that have stylistic similarities (Table 1). F refers to texts that share the same facilitator but no user with stylistic similarities. IU refers to texts that share the same user. RU refers to texts that just share user with stylistic similarities. NR refers to texts that do not share users or facilitators.

A first look at Table 2 shows how the number of texts considered for each group is highly unbalanced. In particular, the group that does not acknowledge any relationship between texts (NR) represents more than half of the samples. Following the order in which the groups appear in Table 2, note that the mean distance value increases as the co-operational effort within texts decreases. The more the stylistic fingerprint is shared (FU) the closer the texts are.

We first carried out a one-sample t-test to evaluate each group's divergence from an average (*μ* = 1,04) or neutral (*μ* = 1) distance score (Table 2). Results show that, on all occasions, groups differentiate from the neutral value (*μ* = 1). Groups FU, F and IU having lower values and groups RU and NR higher values. Similarly, the groups' distance values statistically differ from the average distance registered in the analysis. The only exception is represented by the group RU.

Next, we conducted paired *t*-tests to check whether the inter-group distances are statistically significant. In particular, we focused on the statistical comparison of group IU with all the other groups. To avoid the lack of balance in the number of textual distances for each group, the *t*-test was conducted with a sampled number of distance values and iterated 10 times. The analysis (displayed in Table 3) shows that the registered distance values between the IU group and the others are statistically different (*p* < 0.005) with the only exception being the distance values between texts that share the same user (IU) and texts that share just the same facilitator (F).

**Table 3:** Paired *T*-test (10-time iterated sampling).

Paired *T*-test (10-time iterated sampling)					
Group 1	Mean	Group 2	Mean	Test result	*p* value
IU	0.89	FU	0.78	1	0.0000
IU	0.89	F	0.89	0	0.6281
IU	0.89	RU	1.04	1	0.0000
IU	0.89	NR	1.10	1	0.0000

The mean distance between the IU group and the others is tested with Student's *t*-test). Given the unbalanced number of texts representing each group, the *t*-test was conducted with sampled values in order to equalize the number of observations for each group. Random sampling was conducted ten times. Then the *t*-test was repeated with each sample. The average *p*-value is reported: the registered differences in distance values are statistically significant, with the only exception being the differences between texts that share the same user (IU) and texts that just share the same facilitator (F).

Finally, we used a supervised machine-learning analysis to investigate the number of texts that could be classified correctly according to their group.

**Figure 9:**
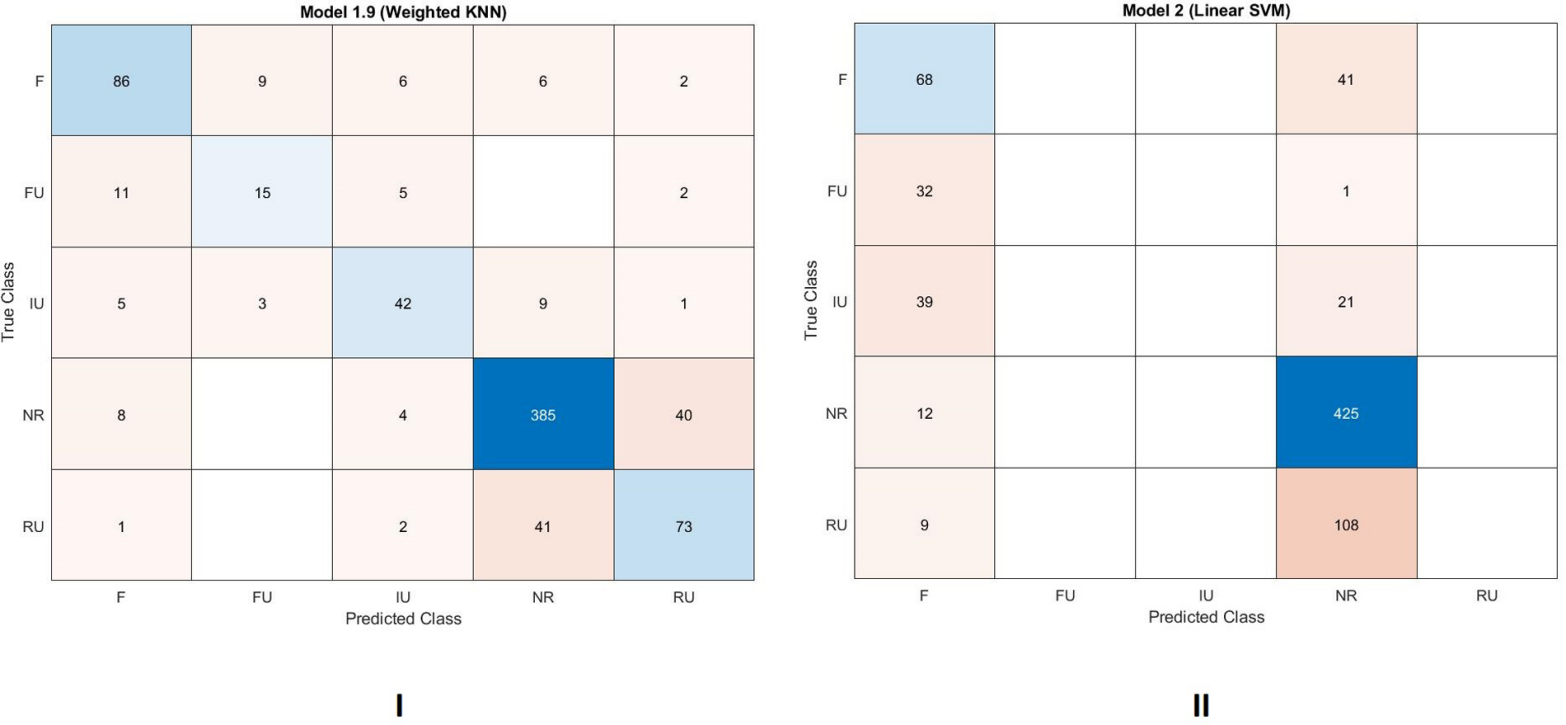
Supervised machine learning analysis. (**I**) Weighted KNN classification, 5-fold cross validation. Accuracy scores 79.49%. Values were assigned to all the five groups. 50 texts that share the same user (83%) are classified as either texts that share the same user or texts that share the same facilitator. F = texts that share the same user but no user with similar stylistic fingerprint. IU = texts that share the same user. FU = texts that share the same facilitator and user with stylistic fingerprint. RU = texts that share user with similar stylistic fingerprint. NR = texts that do not share any user nor facilitator. (**II**) SVM classification, 5-fold cross validation. Accuracy scores 65.21%. Values were assigned to just two different groups. 39 texts that share the same user (65%) are classified as texts that share the same facilitator. F = texts that share the same user but no user with similar stylistic fingerprint. IU = texts that share the same user. FU = texts that share the same facilitator and user with stylistic fingerprint. RU = texts that share user with similar stylistic fingerprint. NR = texts that do not share any user nor facilitator.

The data were tested twice; first, with a weighted KNN algorithm (5-fold cross validation), then with an SVM algorithm (5-fold cross validation). The results are reported in Figures 9I,II. The weighted KNN classification had a 79.49% accuracy and could assign texts distances to all the groups. The SVM classification had a lower accuracy score 65.21% and could assign texts just to 2 groups (F and NR). However, besides the accuracy score, it is worth noting the number of texts that share the same user being classified as texts that share the same facilitator (39 out of 60). A value very similar to that detected in the Bootstrap consensus network analysis (see study 2B below), and a higher value if we considered the rank analysis displayed in Figure 8, where just 27 texts were classified above the red line.

Overall, the statistical analyses conducted with the distance values confirm what was displayed by the rank analysis, namely that the style of texts is influenced either by the facilitator they share or by the user. Moreover, these analyses could positively acknowledge inter-user similarities, reinforcing the presence of user contribution to text creation. While considering these analyses, it should be noted that distance scores are strongly affected by the unbalanced nature of the corpus. The heavy representation of F1, for example (half of the words written in the corpus are assisted by F1), makes F1 the strongest stylistic force detectable in the corpus, impacting consistently even in the choice of the most frequent words and most frequent characters. In addition, the lack of balance in the sizes of texts written by users has an impact on the distance values, not in terms of absolute frequency of some words, but rather in the representativeness of certain words within smaller texts. Let's consider, as an example, the case of U14. He typed more than 64,000 words with F1 and slightly less than 6,000 words with F5. This size imbalance can impact considerably on the distance values between texts as the number of words shared by the two texts may be fewer than those shared by texts with a higher number of words.

### 4.4. Study 2b: bootstrap consensus network analysis

**Figure 10:**
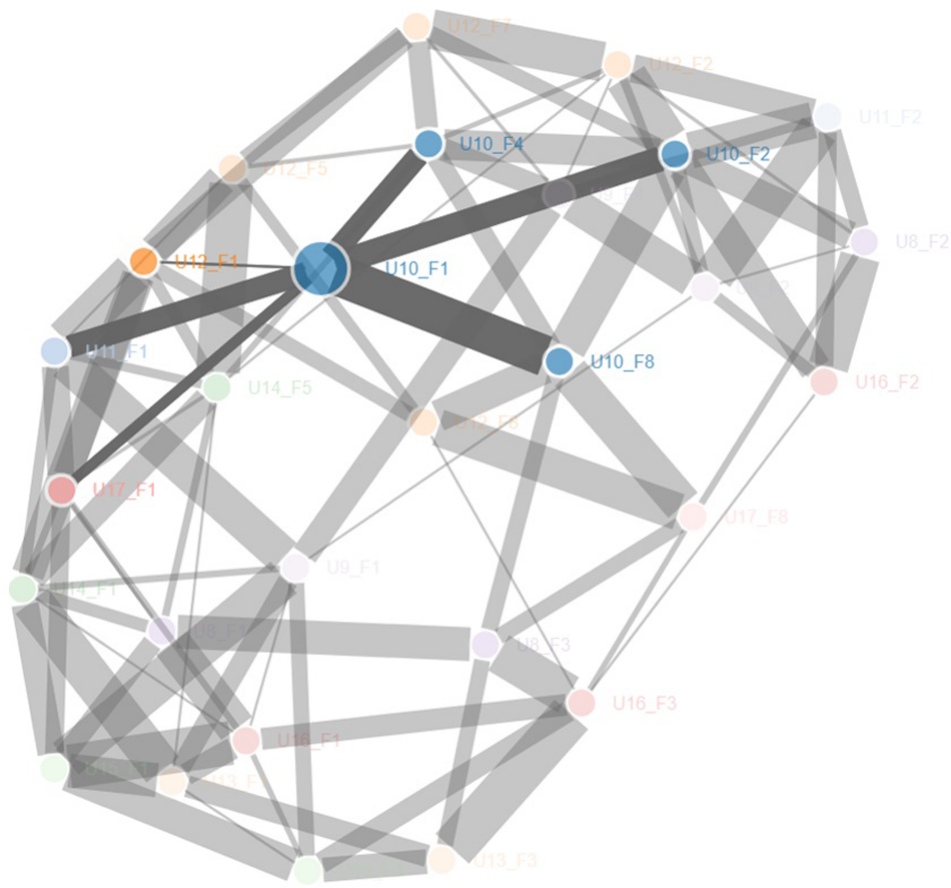
Bootstrap consensus network: graphical display. Nodes represents texts, linkage represents stylistic similarities between texts. The thickness of the linkage is directly proportional to the strength of the similarity. The more the linkage is thick the more the texts that it connects are similar. In the figure above the focus is on U10_F1 and its link with U10_F2, U10_F4, U10_F8, U11_F1, U11_F1 and U17_F1.

**Figure 11:**
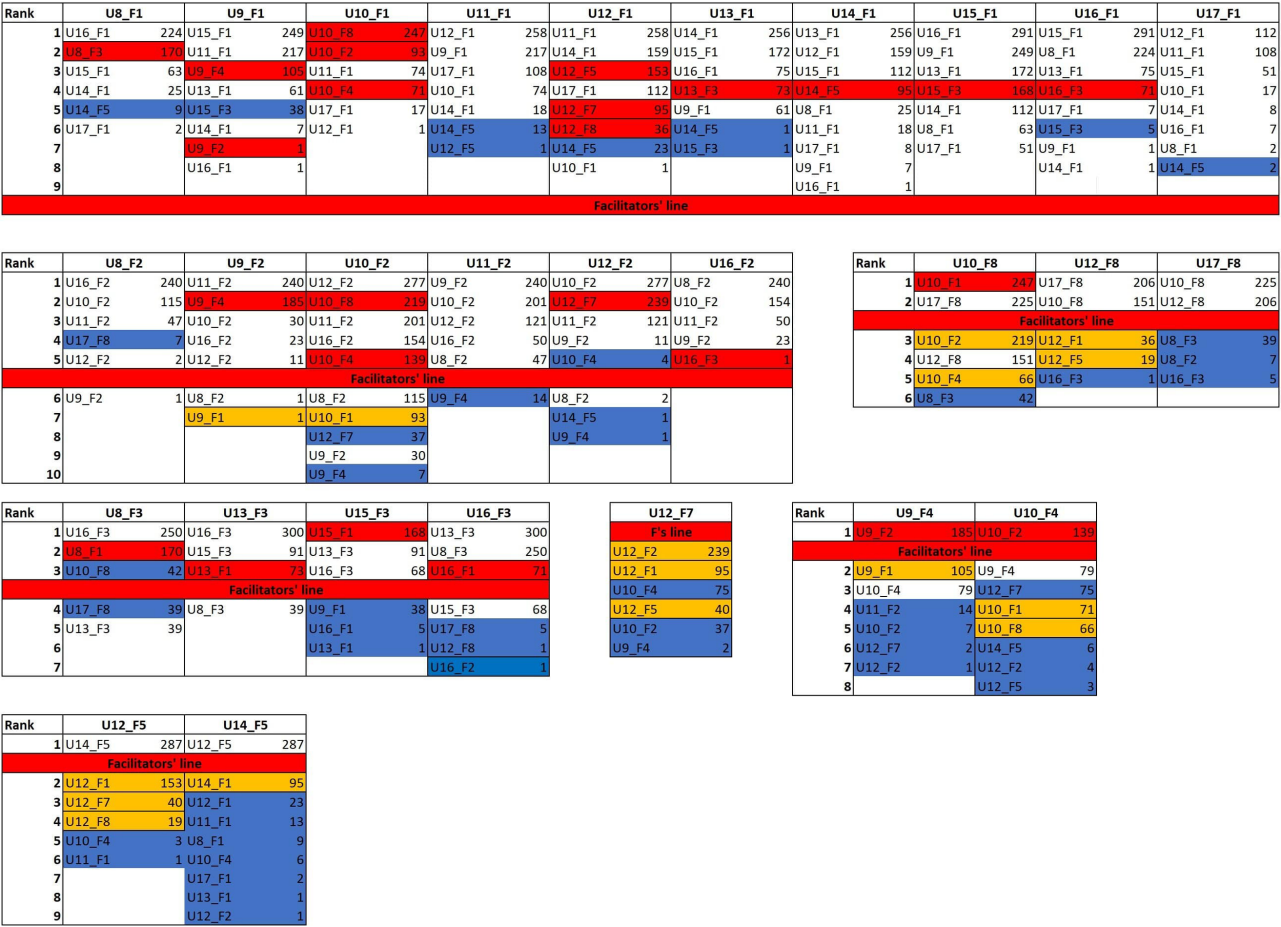
Bootstrap consensus networks: undirected links and weight table. Range: 100 to 5,000 most frequent words. The table represent for each text the links established with the other texts in the corpus and the strength of their relationship, also named weight. Texts are grouped by the facilitator shared; the red line indicate the limit above which we should find just texts written by the facilitator expressed by the column. Red code is used for those texts of the same user expressed by the columns that rank above the red line. As seen in Figure 7, the red line is given by the number of texts written with the same facilitator as the one expressed by the column. So, in the case of column U10_F1, U10_F8, U10_F2 and U10_F4 rank above the red line, respectively at 1st 2nd and 4th position. As for U10_F1 the red line is defined at position 10 all these texts are red coded. These ranks provide a meaningful hint of how texts written by U10 are inherently similar independently of the facilitator involved in the communication. Texts of the same user expressed by the column that rank below the red line are reported in orange. The orange coding is particularly meaningful for those facilitators (as F4, F5, F7 and F8) where the red line is particularly high placed (above the third rank position). 23 texts (out of 60) rank above the red line. 25 texts (out of 60) rank in the first three rank positions. 38 texts (out of 6o) rank in the first five rank positions.

The analysis with the bootstrap consensus network computes and graphically displays all the linkages existing between each text (Figure 10). The consensus network is composed of nodes (that represent each text candidate in the corpus) and undirected links. Two nodes are connected when they show stylistic similarities. The weight of this similarity is proportional to the thickness of the link in the graph. Stylistically similar texts have thicker linkages in the graph. Together with the graphical report of these relationships (Figure 10) the R package returns a hedge table where, for each node, undirected links and their weight are reported (see Figure 11). As before, this table was investigated to address the hypothesis that facilitators are the sole contributors to the production of texts. The color coding is the same as the one adopted for the cluster analysis distance tables. A blue code is added to highlight texts that share neither the facilitator nor the user with the node.

Twenty-five texts rank above the red line, 23 texts classify in the first three positions and 38 in the first 5 positions. Thus, texts of the same user consistently occupy higher ranking positions than would be expected if users did not make a stylistic contribution, and very often they are in the closest neighbourhood of similarity. It should be also noted that of the 190 links recognized by the analysis, 100 out of a possible 140 are between texts that share the same facilitator. The count of 140 refers to the total number of possible combinations of links that can be established between texts written with the same facilitator. For example, since ten users wrote with F1, each text written with F1 can be linked, in theory, to nine other texts written with F1 (thus, 90 links can be established for F1), 30 for F2 (6 users with 5 links each), 12 for F3 (4 users with 3 links each), 2 for F4 and F5 (2 users with 1 link each), and 6 for F8 (3 users with 2 links each). Thus, 42 out of a possible 60 links are between texts that share the same user, and 48 out of a possible 554 links are between texts that share neither the facilitator nor the user. However, if the weight of each link is considered (the higher the weight, the closer the texts), then 96% of the weights of the links are saturated by user-to-user and facilitator-to-facilitator links (27% and 69%, respectively). Moreover, if we consider the average weight for each class (user-to-user, facilitator-to-facilitator, and user-to-facilitator) we see that relationships between texts of the same user, and between texts that share the same facilitator, have a similarly high weight average, while texts that do not share either the facilitator or the user have a lower weight average (see Table 4). Finally, it should be noted how even in the consensus network analysis inter-user similarities can be detected. Relationships are mostly created between the same array of users, independently of the facilitator involved in the communication process. U8 is linked across different facilitators to U16, and U10; U9 to U11 and U10; U10 to U12; U11 to U9, U12, and U10; U12 to U11, U14, and U10; U13 to U15, and U16; U14 to U13, and U12; U15 to U16, U9, and U13; U16 to U13, U8, and U15; U17 to U12, and U10. These links are consistent with the inter-user similarities found in the analysis of the distance tables (Figure 9).

**Table 4:** Bootstrap consensus network: links and weight analysis.

	N° of links	Total weight	Weight %	Average
Same user	42	4,571	27%	109
Same facilitator	100	11,554	69%	115
Different U-F	48	674	4%	14

Statistic regarding links between texts of the same user, between texts written with the same facilitator and links between texts that shared nor the user nor the facilitator are reported. The number of links column counts for each group the number of existing relationships within the corpus. The total weight column indicates for each group the sum of the strength of each existing link. The weight percentage column reports for each group how much the weight strength impact on the analysis. The average column report for each group the average weight that characterizes links. While links between texts of the same user are fewer than all the other possible link, their weight account for the 27% of the total. The average weight existing between texts of the same user is quite similar to the one that exists between texts written with the same facilitator. Interestingly, this table shows that the bootstrap consensus network is able to detect similarities between texts that share the user or the facilitator.

### 4.5. Summary

This study was designed to test the assumption that FC users do not contribute stylistic characteristics to the texts they produce with the assistance of multiple facilitators. If this was the case, cluster analysis and bootstrap consensus network results would not show any grouping between texts of the same user, nor any pattern of similarity between texts of the same user. Cluster analysis results were not consistent with this assumption. The cluster analysis conducted on texts divided by user-facilitator pairs (Figures 6I,II, 7,8) shows how relationships are detected mainly between texts that share either the user or the facilitator. This claim is also supported by the bootstrap consensus network analysis (Figures 9–11) that shows that two major stylistic forces can be ascertained: that of the facilitator and that of the user. If facilitators were the only authors of texts, no significant similarities between texts of the same user across different facilitators should have been observed. Clearly, these results are not consistent with the starting assumption. Besides, there were also patterns of inter-user similarity, consistent with what was shown in Study 1. The existence of these similarities also refutes the starting hypothesis.

## 5. General discussion

The reported studies challenge the maximally sceptical starting position, namely that the facilitator is the sole agent to whom text written by FC users can be attributed. The results clearly show that texts written via FC generally present two linguistic imprints: the user and the facilitator. Based on this result, FC is better described as a co-creation process in which two distinct and active participants collaborate in the production of linguistic content. It is worth noting that this acknowledgement of dual linguistic imprints cannot fully account for the nuances of the text production dynamic of FC. The nature of the corpora and these methods do not allow us to discern the details of what originates with the user and the facilitator. The scope of these analyses is at the stylistic level with no presumption nor power to distinguish facilitators' traits from users' features on a sentence-by-sentence basis. Also, these analyses do not allow any comment on how the facilitator influences and supports the user. These questions require other methods and task-oriented analyses. What the present results clearly show is that the user is not linguistically passive, and the facilitator moulds rather than wholly constructs the typed text.

Acknowledging two stylistic forces in the production of FC text does not shed light on the nature of the contents expressed in the texts. Co-authorship does not offer certainty that a message written by integrating two linguistic sources fully mirrors the FC user's own intention. The existence of user-dependent similarities at the lexical level suggests, however, that users are actively involved in the selection of lexical forms, the linguistic components specifically deputed to convey meaning. This demonstrates the FC users' ability to transform semantic concepts into linguistic code, coherently within given syntactical and pragmatic contexts. This suggests that FC users possess a level of literacy skills, which any AAC intervention should seek to nurture and develop to improve users' quality of life.

It could be argued that an AAC technique that cultivates co-authorship may not foster the users' autonomy and independence. This issue of autonomy, alongside that of authorship, has often been central to the debate on the utility of FC. These two issues should be considered and addressed separately as they may reflect two different goals. This research addressed the issue of authorship and the possibilities of increasing the AAC users' communication options. The results clearly show that a technique like FC extends users' ability to express themselves linguistically. A co-authorship framework does not guarantee independence, which may reach different levels in the case of different users. These differences may originate in the users' inherent characteristics or in the stage of development in FC training. Over time, significant autonomy of expression may be achievable for some, but not for others. In both cases, however, using an AAC technique fostering co-authorship may provide developmental and quality of life advantages through increased communication options that might not otherwise become available.

Finally, it is important to consider the extent to which stylistic characteristics may be consciously or unconsciously modified or adapted in a collaborative setting such as FC. Stylometry and authorship attribution models are built on the general assumption that each person possesses a unique and unconscious stylistic fingerprint that can be detected and quantified through statistical procedures. This notion, first introduced by (51) is widely held in the field of stylometry (41, 52) as it has been proven valid in multiple authorship attributions. However, the possibility that authors can directly manipulate their own style for privacy or falsification has been addressed in recent times. For example, Brennan et al. (53) showed how non-expert users can obfuscate their own stylistic fingerprint or imitate the one of a given model. To what extent the results provided in this paper can be interpreted as the effect of facilitators' style imitation requires comment.

It could be argued that user-dependent stylistic similarities detected in Study 1 and 2, are nonetheless the results of facilitators modifying their style to render it more attributable to the FC user. All studies conducted so far on adversarial stylometry have dealt with conscious and deliberate style modification (53). Suggesting that facilitators deliberately modify their own style to suit the user they are working with would imply that the facilitators' influence on the generated text is conscious. The sceptical position on this has been that the facilitators' influence is an unconscious ideomotor effect (12, 15). This contradiction must be addressed if an imitation hypothesis is adopted. Moreover, no studies have so far demonstrated the possibility of imitating more than one style simultaneously. This hypothesis, while logically possible, requires demonstration. Besides, even if we assume that facilitators consciously manipulate their style such that statistical models end up attributing texts to users instead, and also that facilitators can maintain up to ten different stylistic systems and use them in the correct contexts, we must contend with the issue of stylistic models. Style imitation research has always dealt with participants that deliberately conform their style to that of a model (53–55). However, in the case of FC, facilitators have no external models to which they can refer, so it is unclear on which basis they would organize their imitation. The users would have to have their own styles for the facilitator to imitate and mix with their own. It is unclear how these styles would become known to the facilitator if the users can only express themselves through collaboration with the facilitator in the first place.

It could still be argued that a model is created by the first or most consistent user-facilitator pairing. The first facilitator of each user may develop a style that obfuscates their own and has some unique and individual characteristics that individuate the user, as the results of Study 1 may suggest. Other facilitators who work with the user later might imitate the style developed by the first facilitator for that specific user. If this is how FC develops, we would expect that the imitation would reflect all the stylistic characteristics of the produced text, including those that are due to the facilitator. The results of Study 2 show that independent stylistic signatures are detectable for the facilitators and user. It is unclear how a later facilitator can purposely select user-dependent characteristics in their imitation, but leave out those of the original facilitator, especially at the level of word and character-trigram frequency. This would require discriminating patterns that are facilitator-dependent from those that are user-dependent, and only mixing the user-dependent ones with their own style. This would need to be done separately for every user with whom the facilitator works. It is highly unlikely that such stylistic imitation is feasible. A more likely, and certainly more parsimonious, explanation of the results obtained here is that both the facilitator and user contribute to the style of jointly produced text.

One possible dynamic underlying the observed co-authorship is that facilitators create syntactical structures within which users can fill in their own content. Whether this form of scaffolding occurs would need to be investigated using qualitative analysis. Syntactical scaffolding by the facilitator may also involve adjusting morphological endings, suggesting linkers or auxiliary verbs, or providing syntagmatic prompts to help begin communication (e.g, “I think that …”, or “I feel that …”). These prompting actions could be unique to each facilitator, which could lead to algorithms recognizing text with these expressions as authored by facilitators. In this respect, the facilitators' work would be comparable to that of editors. As described in the case of Hildegard of Bingen (56), the editorial effort of adjusting sentences online (Hildegard could not write so she dictated her thoughts) can lead to algorithmic detection of the stylistic fingerprint of the editor, very much like the present results obtained for FC facilitators. It should also be noted that no redactional revision of the texts was performed. In fact, even in the choice of particular redactional forms can reside clues of someone's participation in the typing process. For example, the Italian word corresponding to /yes/ was used in the text in two alternative forms: the accented and correct one /sì/ and the clitic /si/, incorrect, though used habitually in colloquial writing. The use of the first version appears consistently throughout the texts written with F2, while the second one among the same users when writing with F1. Although no change in meaning is conveyed by choosing alternate one of the two forms, this facilitator-dependent discrepancy contributes to enhancing the distance between texts of the same user and in approaching texts that share the same facilitator. Given the modality of data collecting that -followed a retrospective approach (since it facilitated the collection of texts of greater size), there was no chance of deciding whether these redactional differences originated from the natural flow of FC dynamics, and therefore the facilitator's direct influence, or rather were the result of an explicit, post-hoc corrective intervention from the facilitator. This aspect represents a limitation of the methodology, so future studies should consider controlling possible external redactional interventions, being cautious to not interfere with the natural dynamic of FC.

If the presented evidence of FC users’ stylistic contribution is accepted, consideration shifts to the utility of an AAC technique that may enable the user to co-author texts with trained facilitators. Clearly, a technique that, in due course, leads demonstrably to independent text production ought to be preferred over one that involves dependence on the assistance of a trained facilitator who also co-authors the user's texts. The issue of assisted communication only arises, however, in the context of significant sensorimotor and cognitive disabilities. Depending on the level of these disabilities, developing towards autonomous linguistic expression may not be a feasible goal. What must be judged in such cases is the potential utility of providing the individual with an effective means of text co-production that may not ever result in autonomy. The present results clearly demonstrate that the latter option does result in the individual producing a personal linguistic signature, even though this happens only with the availability of co-productive assistance. Given the limited prospects of these individuals conducting any aspects of their lives autonomously, and their expected dependence on others' care for the rest of their lives, we submit that co-creating text using a technique such as FC is very likely to be a developmentally and psychologically valuable exercise.

## 6. Limitations and future directions

There are several limitations to the work reported in this paper, and these are mostly inherent in the corpus of text that was produced over an extended period of time (>10 years in some cases) and was not designed in any way for the requirements of research. First, the available corpus is inherently unbalanced in the number of words representing each user and facilitator, and in the user-facilitator pairings. For instance, some users in the corpus have written consistently with five different facilitators while some others with just two. As we have noted, an effect of this imbalance is that some stylistic fingerprints, particularly those of the most prolific facilitators, are more consistently represented than others. Controlled corpus construction would aim for a more homogeneous assortment of user-facilitator pairs, with all users sharing the same set of facilitators, and writing a similar number of words in each user-facilitator pair. Such a controlled corpus comparable in size to the one analyzed here would require many years to accumulate.

Second, adopting a historical corpus precluded any control over the production of textual content. It was not possible to ascertain direct or indirect redactional interventions that could have affected the strength of textual similarities. It was also impossible to control the topics that were addressed in the generated text. On one hand, the text analysed is thereby free of the demand characteristics of a research study, but on the other, linguistic analysis is impacted by the divergence of topics chosen by facilitators or users, leading to uncontrolled differences in lexical targets.

A third limitation arises from the heterogeneity of cognitive and sensorimotor function among the users represented in the corpus, and the differences in their age, DD diagnosis, levels of facilitation, and the period of FC use. In addition, it was not possible to collect information about the level of the users' language comprehension or literacy. Thus, the only confirmed commonalities among the users were their adoption of FC and the facilitators they had shared.

Finally, despite the long periods over which the corpus was produced, the present methods did not allow us to look for a developmental trajectory in the users' stylometric contributions to their texts. Future studies should aim to investigate whether the user's stylistic fingerprint changes strength with increased experience with FC, and if so, which conditions of training or practice best facilitate this.

## 7. Conclusion

The studies presented in this paper analysed a large corpus of FC text produced over a number of years to test the hypothesis that only the facilitator's stylistic signature should be detectable in the text. This hypothesis follows from the view that the facilitator is the sole author of FC text. The results do not support this hypothesis as the user's stylistic fingerprint is detectable alongside those of facilitators. The conclusion is that FC text should be viewed as co-authored by the user and the facilitator. As the user is clearly a participant in text generation, there is scope for touch-based assistance to serve as a scaffold in DD individuals' linguistic development, and to contribute positively to their quality of life and connection with carers. Whether the individual does, or could develop to, generate typed text independently should not determine the value of practising and better understanding touch-assisted typing techniques (9). Future work on these techniques should instead establish a developmental context within which research focuses on how best to utilize these techniques to enhance DD individuals' education and well-being. Such an approach would recognize the full significance of this paper's findings. Just as the present analysis has shown that the FC user is not a passive recipient of ideomotor suggestion, it has also shown that the facilitator actively shapes the text that is typed. The level of facilitator contribution we have reported advises us that, in the proposed developmental approach, uses of FC texts should always be informed by the co-creative nature of the process.

## Data Availability

Publicly available datasets were analyzed in this study. This data can be found here: https://doi.org/10.5281/zenodo.7273147.
